# Chromosome 9p21 gene copy number and prognostic significance of *p16* in ESFT

**DOI:** 10.1038/sj.bjc.6603819

**Published:** 2007-05-29

**Authors:** S C Brownhill, C Taylor, S A Burchill

**Affiliations:** 1 Candlelighter's Children's Cancer Research Laboratory, St. James's University Hospital, Beckett Street, LS9 7TF Leeds, UK; 2 Mutation Detection Facility, St. James's University Hospital, Beckett Street, LS9 7TF Leeds, UK

**Keywords:** ESFT, MLPA, p16, p14^ARF^, prognosis

## Abstract

Chromosome 9p21 gene copy number in Ewing's sarcoma family of tumour (ESFT) cell lines and primary ESFT has been evaluated using Multiplex Ligation-dependent probe amplification, and the clinical significance of *CDKN2A* loss and p16/p14^ARF^ expression investigated. Homozygous deletion of *CDKN2A* was identified in 4/9 (44%) of ESFT cell lines and 4/42 (10%) primary ESFT; loss of one copy of *CDKN2A* was identified in a further 2/9 (22%) cell lines and 2/42 (5%) tumours. *CDKN2B* was co-deleted in three (33%) cell lines and two (5%) tumours. Co-deletion of the *MTAP* gene was observed in 1/9 (11%) cell lines and 3/42 (7%) tumours. No correlation was observed between *CDKN2A* deletion and clinical parameters. However, co-expression of high levels of p16/p14^ARF^ mRNA predicted a poor event-free survival (*P*=0.046, log-rank test). High levels of p16/p14^ARF^ mRNA did not correlate with high expression of p16 protein. Furthermore, p16 protein expression did not predict event-free or overall survival. Methylation is not a common mechanism of *p16* gene silencing in ESFT. These studies demonstrate that loss (homozygous deletion or single copy) of *CDKN2A* was not prognostically significant in primary ESFT. However, high levels of p16/p14^ARF^ mRNA expression were predictive of a poor event-free survival and should be investigated further.

The Ewing's sarcoma family of tumours (ESFT) is defined by the presence of an *EWS-ETS* gene rearrangement involving the *EWS* gene on chromosome 22q12 with one of a number of *ETS* genes; most frequently with *FLI1* on chromosome 11q24 (Turc-Carel *et al*, 1983; Delattre *et al*, 1994). These gene rearrangements generate aberrant transcription factors that are important in the development and maintenance of the ESFT malignant phenotype (May *et al*, 1993; Teitell *et al*, 1999; Rorie and Weissman, 2004). However, up to 80% of primary ESFT have nonrandom secondary chromosome changes (Mugneret *et al*, 1988; Kullendorff *et al*, 1999; Zielenska *et al*, 2001), several of which are reported to be prognostically significant.

Loss of the cell-cycle regulatory gene *CDKN2A* exon 1*α* (which codes for p16) has been reported in between 13 and 30% of ESFT (Kovar *et al*, 1997; Tsuchiya *et al*, 2000; Wei *et al*, 2000; Huang *et al*, 2005). *CDKN2A* exon 1*β* (p14^ARF^) is often co-deleted with *CDKN2A* exon 1*α* (Kovar *et al*, 1997; Tsuchiya *et al*, 2000; Wei *et al*, 2000). Loss of p16 protein expression has also been reported in a small panel of ESFT (Maitra *et al*, 2001), although the prognostic significance of this loss remains unclear. Although homozygous deletion of *CDKN2A* exon 1*α* has been described, the frequency and impact of single gene copy number changes has not been investigated.

Gene copy number in up to 45 nucleic acid sequences can be analysed using multiplex ligation-dependent probe amplification (MLPA; Schouten *et al*, 2002) and has been successfully exploited to identify constitutive gene copy number changes in many genes including the *BRCA1* gene in breast and ovarian cancer families (Hogervorst *et al*, 2003; Montagna *et al*, 2003; Bunyan *et al*, 2004; Hartmann *et al*, 2004) and the *MSH2* and *MLH1* genes in patients with Lynch syndrome (Gille *et al*, 2002; Nakagawa *et al*, 2003; Taylor *et al*, 2003; Bunyan *et al*, 2004). More recently, the technique has been applied to the analysis of copy number changes in tumours (Aveyard and Knowles, 2004).

The aims of this study were to investigate the frequency of homozygous and single copy loss of *CDKN2A* and other genes located on chromosome 9p21 in primary ESFT. The methylation status of the p16 promoter was also assessed, and the prognostic significance of *CDKN2A* loss, p16/p14^ARF^ mRNA and p16 protein expression in primary ESFT investigated.

## MATERIALS AND METHODS

### Cell lines and clinical samples

Nine ESFT cell lines (A673, RDES, TC-32, TTC-466, SK-N-MC, SKES1, GROH1, STAET1 and STAET11), three neuroblastoma (IMR-32, SK-N-SH and SH-SY5Y) and one colorectal carcinoma (SW480) cell lines were investigated. TC-32, RD-ES, A673, SK-N-MC, SKES1, TTC-466, SH-SY-5Y and SK-N-SH cells were cultured as described previously (Myatt *et al*, 2005). GROH1, STAET1 and STAET11 were grown in RPMI 1640 (Sigma, Dorset, UK) containing 10% fetal calf serum (FCS; SeraLab, Sussex, UK), SW480 cells in Dulbecco's modified eagle's medium (DMEM; Sigma) and 10% FCS and IMR-32 cells in 1:1 DMEM:RPMI 1640. GROH1, STAET1 and STAET11 cells were kind gift from Dr H. Kovar (Children's Cancer Research Institute, Vienna, Austria).

Diagnosis of ESFT (*n*=42) was confirmed by reverse transcriptase (RT)-PCR for the *EWS-ETS* gene rearrangements (36/42) or immunohistochemistry for MIC-2 (CD99; 6/42). The presence of metastases were detected by conventional imaging and examination of bone marrow by light microscopy. Informed consent was obtained for the use of tumour material for biological studies. Ethical approval was obtained from the Leeds NHS Trust Ethics Committee (MREC 98/0/44) and the CCLG tumour bank (MREC 98/4/023).

### Extraction of DNA and RNA

Total RNA was extracted from cell lines using Ultraspec™ (Biotecx; Houston, TX, USA; Burchill *et al*, 1994), and DNA using the DNeasy Tissue kit (Qiagen, West Sussex, UK) according to manufacturer's instructions.

Frozen sections of primary ESFT were stained with haematoxylin and eosin and tumour cells isolated by laser-capture micro-dissection, using the PixCell™ laser-capture micro-dissection system (LCM; Arcturus, Sunnyvale, CA, USA). DNA and RNA were extracted from the isolated tumour cells using the QIAamp DNA Micro kit (Qiagen) and the PicoPure™ RNA Isolation Kit (Arcturus), respectively.

The quantity and quality of RNA and DNA were measured by assessment of absorbance at 260 and 280 nm (Nanodrop, Labtech International; Ringmer, East Sussex, UK).

### Multiplex ligation-dependent probe amplification

Multiplex ligation-dependent probe amplification was performed on DNA diluted in TE buffer (5 *μ*l; 10 mM Tris–HCl (pH 8.2) and 1 mM EDTA), using the 9p21 MLPA SALSA P024 CDKN2A/2B region Deletion Test Kit (MRC Holland; Amsterdam, The Netherlands) according to manufacturer's instructions. Bovine serum albumin (1 *μ*g; Sigma) was added to DNA from LCM tumour samples to improve amplification efficiency. DNA from cell lines (200 ng) was amplified for 33 cycles and from tumours (21–201 ng) for 36 cycles on the GeneAmp PCR System 9700 thermal cycler (Applied Biosystems, Warrington, UK). Data was analysed using the ABI PRISM® 3100 Genetic Analyser and POP4 Genescan polymer, and MLPA data collected using Genescan software (Applied Biosystems). Peak heights of each probe were compared to those of the average of two normal control samples (previously determined to be wild type for all of the genes tested) to obtain a dosage quotient (DQ). The DQ represents the gene dosage of each probe and was calculated using the following formula:

DQ=((*A*/*B*)/(*A*′/*B*′)) where *A* is the peak height of the patient test probe A, *B* is the peak height of the patient internal control B and *A*′ and *B*′ are the peak heights of the same probes from the average of the normal control samples. The DQ was calculated for each test probe in comparison to each internal control probe and the mean average DQ for each test probe calculated. The standard deviation of the DQ of a test probe compared to each internal control probe was also calculated. A DQ of 1=both copies of the gene are present, DQ=0.5 one copy of the gene is deleted, DQ=>1.5 is indicative of gene duplication and DQ=0 both copies of the gene are absent.

### Methylation-specific PCR

DNA (500 ng cell line, 100 ng of primary ESFT) in a total volume of 45 *μ*l was treated with the EZ DNA Methylation Kit (Cambridge BioScience, Cambridge, UK) and eluted into 10 *μ*l of M-Elution Buffer. Methylation-specific PCR was performed for the p16 promoter region on cell lines and tumours without homozygous deletion of *CDKN2A*. Modified DNA (2 *μ*l cell line, 4 *μ*l primary ESFT) was amplified in a 50 *μ*l PCR containing 300 ng of sequence-specific primers (Herman *et al*, 1995), 0.2 mM dNTPs (Amersham Biosciences, Buckinghamshire, UK), 1.5 mM MgCl_2_ (Sigma) and 1 U of AmpliTaq Gold DNA polymerase in 1 × AmpliTaq Reaction Buffer II (Applied Biosystems). Amplitaq Gold was activated by heating (95°C × 10 min), and DNA amplified using 35 cycles of 95°C × 30 s, 60°C (unmethylated PCR) or 65°C (methylated PCR) × 30 s and 72°C × 1 min, with a final extension of 72°C × 4 min. PCR products were separated on a 2% agarose gel, stained with ethidium bromide (0.5 *μ*g/ml) and visualised under ultraviolet light.

### Real-time RT-PCR

RNA (250 ng cell line, 5 ng primary ESFT) was reverse-transcribed with 5 U of murine leukaemia virus RT in 8 U of RNA guard, 1 mM dNTP, 8 mM MgCl_2_, 0.3 *μ*g random hexamer primers and 1 × PCR buffer (10 mM Tris–HCl (pH 8.3), 50 mM KCl; Applied Biosystems) made up to a total volume of 5 *μ*l with diethyl pyrocarbonate (DEPC)-treated H_2_O. The resulting cDNA was added to 20 *μ*l of PCR mix containing a final concentration of 1 × TaqMan Universal PCR Master Mix (containing AmpliTaq gold DNA Polymerase; Applied Biosystems) with 1 × CDKN2A TaqMan Gene Expression Assay (this assay contains primers and probe to amplify exons 2 and 3 of the *CDKN2A* gene encoding both p16 and p14^ARF^ mRNA; Applied Biosystems) or primers (100 nM) and TaqMan probes (100 nM labelled with 5′-FAM™ and 3′-TAMRA™) to amplify *β*_2_-microglobulin mRNA (*β*_2_M) in DEPC-treated H_2_O.

*β*2M forward primer 5′-GAGTATGCCTGCCGTGTG-3′,

*β*2M reverse primer 5′-AATCCAAATGCGGCATCT-3′,

*β*2M probe 5′-CCTCCATGATGCTGCTTACATGTCTC-3′

Samples were amplified in triplicate (1 × 95°C for 10 min, followed by 40 cycles of 95°C × 15 s, 60°C × 1 min) and analysed on the ABI PRISM 7700 Sequence Detector (Applied Biosystems). Negative controls included samples in which template or RT was absent; RNA extracted from the SK-N-MC cell line was included as a reference sample. The fold change in expression was determined using the comparative *C*_T_ method (see www.appliedbiosystems.com). Expression was scored as low or high based on a cutoff of 0.1; this was selected as it corresponded to the upper quartile of the ratios in the data.

### Immunohistochemistry for p16

Frozen tumour from 37 patients at diagnosis was available for immunohistochemistry. Sections (5 *μ*m) were fixed on glass slides for 2 × 2 min in methanol:acetone (50:50), allowed to air dry and expression of p16 examined using a p16 monoclonal antibody (1:1000 for 1 h; Ab-7; Labvision, Fremont, CA, USA) and the catalysed signal amplification system (CSA System; DakoCytomation, Cambridgeshire, UK) according to manufacturer's instructions. Endogenous biotin or biotin-binding proteins were blocked using the Avidin Biotin blocking kit (Vector Laboratories, Peterborough, UK) according to manufacturer's instructions, and endogenous peroxidase activity blocked using 3% hydrogen peroxide in water for 5 min (CSA System). Sections were counterstained with haematoxylin, dehydrated and mounted in DePex mounting medium (VWR International, Leicestershire, UK). p16 expression was examined by light microscopy and scored in tumours as absent (negative), expressed or highly expressed. The percentage of cells positive for p16 was calculated by counting the number of positive cells per 100 cells in three fields.

### Statistical analyses

The prognostic value of *CDKN2A* gene status, p16/p14^ARF^ mRNA and p16 protein expression was evaluated using the log-rank test. Overall survival was calculated as the time from diagnosis to the date last seen, regardless of the number of events that may have occurred. Event-free survival was defined as the time from diagnosis to the time of first event; first event could be relapse, death or date last seen for surviving patients. Associations between *CDKN2A* gene status, p16/p14^ARF^ mRNA expression and clinical features were evaluated using Fisher's exact test. Statistical analysis was performed using the SAS statistical software (SAS Institute Inc., Cary, NC, USA).

## RESULTS

### *CDKN2A* status detected by MLPA in ESFT cell lines

The 9p21 MLPA SALSA P024 CDKN2A/2B region Deletion Test Kit contains 17 control probes, two of which are located on chromosome 8 (8q24 and 8p23). Analysis of the MLPA results for the ESFT cell lines revealed that in 8/9 one or both of the two control probes located to chromosome 8 were duplicated, leading to an inaccurate assessment of the copy number of the 9p21 probes. Cytogenetic analysis identified gain of chromosome 8 in 7/8 of these cell lines (data not shown), consistent with previous literature (Armengol *et al*, 1997; Tarkkanen *et al*, 1999; Hattinger *et al*, 2002). Therefore the control probes on chromosome 8 were excluded when analysing data from ESFT. Gain or loss of additional control probes on 5q, 1p, 7p, 11p, 14q, 5q, 11q, 17p, 7q, 22q, 2p and 10p were not identified across ESFT DNA.

**Figure 1:**
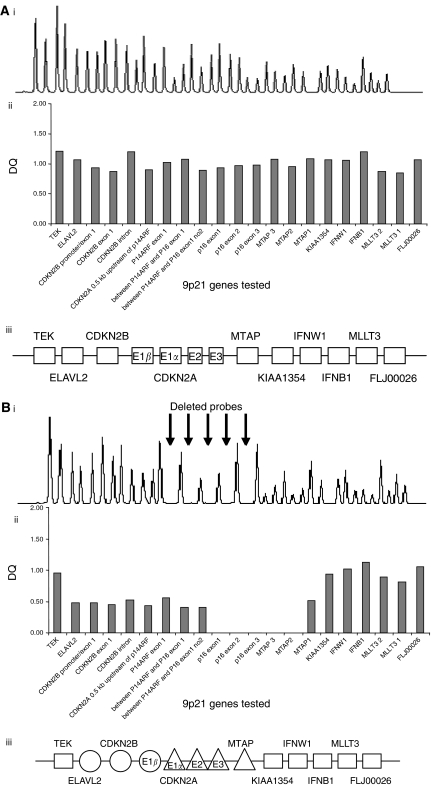

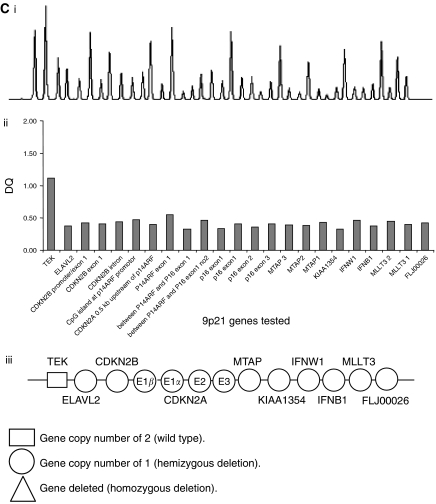
Multiplex ligation-dependent probe amplification analysis of ESFT DNA. DNA was extracted from the (**A**) SK-N-MC, (**B**) TC-32 and (**C**) RDES cell lines and analysed for deletions of genes at the 9p21 region by MLPA. (i) Amplification products were separated by electrophoresis and analysed using Genescan software. (ii) The 9p21 probe peak heights are displayed relative to control probe peak heights to identify gene copy number. The 9p21 genes tested are displayed on the *x* axis and the dosage quotient (DQ) is displayed on the *y* axis. (iii) Diagram showing the gene copy number of genes located at chromosome 9p21, E – exon. The SK-N-MC cell line exhibits a wild-type copy number of all genes tested, MLPA has identified both homozygous and single copy deletion of genes in the TC-32 cell line and the RDES cell line has a single copy deletion of the majority of the genes tested.

All 9p21 genes were wild type in the three neuroblastoma cell lines and 3/9 (33%) of the ESFT cell lines. Homozygous deletion of *CDKN2A* was identified in 4/9 (44%) ESFT cell lines, co-deletion of *CDKN2A* exon 1*β* and *CDKN2B* occurred in three (33%) and co-deletion of *MTAP* in 1/9 (11%). Single copy deletion of *CDKN2A* was identified in further 2/9 (22%) ESFT cell lines, and of further genes in four cell lines (in two of these homozygous deletions had been identified) (Table 1, Figure 1). Deletions of *CDKN2A* were more frequent in ESFT cell lines (44%) than in primary tumour material (10%; see below), consistent with the literature (Kovar *et al*, 1997). This could reflect the selection of tumour cells for growth in culture or accumulation of genetic abnormalities during culture.

### Expression of p16/p14^ARF^ mRNA and p16 promoter hypermethylation in ESFT cell lines

**Figure 2:**
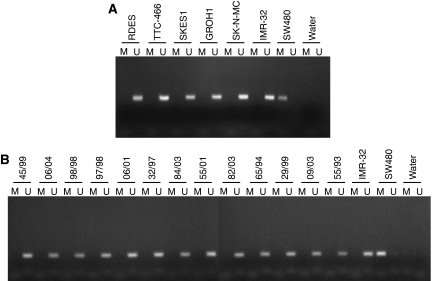
Methylation-specific PCR analysis of the promoter region of p16. DNA extracted from (**A**) ESFT cell lines and (**B**) primary tumour samples was treated with the EZ DNA Methylation Kit and subjected to MSP. The presence of a PCR product in lanes labelled U indicates the presence of unmethylated p16 promoter regions, the presence of a PCR product in lanes labelled M indicates the presence of p16 promoter hypermethylation. Treated DNA extracted from the IMR-32 and SW480 cell lines was used as negative and positive controls, respectively, for p16 promoter hypermethylation.

Hypermethylation of the p16 promoter was not observed in any of the ESFT cell lines where the *CDKN2A* status was wild type or single copy deleted (Figure 2A).

**Figure 3:**
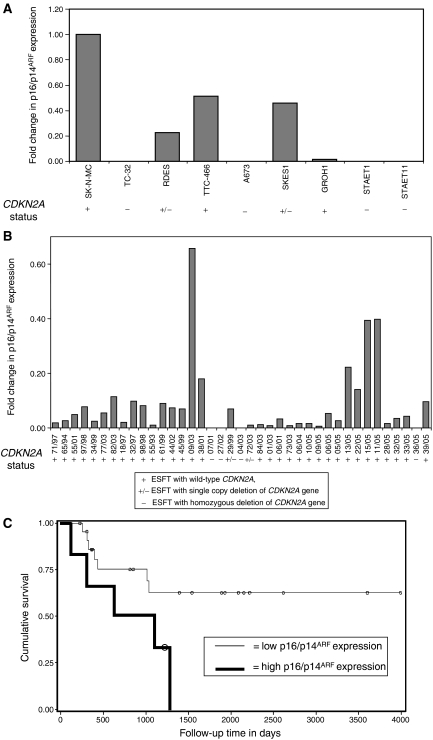
Comparison of p16/p14^ARF^ levels in ESFT cell lines and primary samples. (**A**) RNA was extracted from ESFT cell lines and p16/p14^ARF^ and *β*_2_-microglobulin expression levels were determined by real-time RT-PCR. Expression in each cell line was normalised to expression of *β*_2_-microglobulin and relative to a reference sample (SK-N-MC); expression levels are presented as a fold change in expression. (**B**) RNA was extracted from cells isolated by laser-capture micro-dissection from 5 *μ*m sections of primary ESFT. Expression of p16/p14^ARF^ and *β*_2_-microglobulin was determined by real-time RT-PCR; expression in each tumour was normalised to expression of *β*_2_-microglobulin and relative to a reference sample (SK-N-MC). (**C**) Kaplan–Meier survival plot to compare the event-free survival of patients with tumours that had a high p16/p14^ARF^ mRNA expression level (⩾0.1) to that of patients with tumours that had a low p16/p14^ARF^ mRNA expression level (<0.1), *P*=0.043; log-rank test. Circles define censored events.

Cell lines with homozygous deletion of *CDKN2A* did not express p16/p14^ARF^ mRNA (Figure 3A). Although expression of p16/p14^ARF^ across the cell lines was heterogeneous, there was no correlation with *CDKN2A* copy number (*P*=0.13; analysis of variance (ANOVA) with *t*-test). The reference sample (SK-N-MC) had the highest level of p16/p14^ARF^ expression; this cell line is *p53* null consistent with the hypothesis that p14^ARF^ expression is elevated in p53-deficient cell lines (Nakagawa *et al*, 2003; Myatt *et al*, 2005). p53 was expressed in all other cell lines except the *CDKN2A*-deleted A673.

### *CDKN2A* status in primary ESFT samples

Eighty-six percent (36/42) of primary ESFT were wild-type *CDKN2A*. Homozygous deletion of *CDKN2A* was identified in 4/42 (10%) of tumours, co-deletion of *CDKN2A* exon 1*β* in three and *CDKN2B* in two of these samples. Co-deletion of *MTAP* was observed in 3/42 (7%) tumours. Single copy loss of 9p21 genes was observed in 3/42 (7%) tumours, two of these single copy losses were of the *CDKN2A* gene (5%; Table 2). There was no relationship between *CDKN2A* gene status and overall (*P*=0.42, log-rank test) or event-free (*P*=0.88) survival when patients with homozygous deletion tumours were compared to those that were wild type. No association was observed between *CDKN2A* gene status and the presence of metastatic disease at diagnosis (*P*=0.21, Fisher's exact test).

The median overall and event-free survival for patients wild type for *CDKN2A* was not estimable and 42 months (95% confidence interval (CI)=15−∞), respectively, the median overall and event-free survival for patients with homozygous deletion of *CDKN2A* was 36 months (95% CI=19−∞) and 34 months (95% CI=14−∞), the median overall and event-free survival times were not estimable for patients with single copy *CDKN2A* deletions.

### Expression of p16/p14^ARF^ mRNA, and p16 promoter hypermethylation in primary ESFT

Tumour expression of p16/p14^ARF^ mRNA was heterogeneous (Figure 3B) and although there was no correlation between expression and *CDKN2A* copy number (*P*=0.35; ANOVA with *t*-test), homozygous deletion of the *CDKN2A* gene (samples 07/01, 27/02, 04/03 and 36/05) resulted in loss of p16/p14^ARF^ mRNA expression.

The prognostic significance of p16/p14^ARF^ mRNA expression was assessed in cases (*n*=33), where clinical outcome information was available. The median overall and event-free survival for patients with tumours expressing high levels of p16/p14^ARF^ mRNA (⩾0.1, *n*=6) were not estimable and 872 days (95% CI=296–1290 days), respectively. The median overall and event-free survival times for patients with tumours expressing low levels of p16/p14^ARF^ mRNA (<0.1, *n*=26) were not estimable. No relationship was observed between p16/p14^ARF^ mRNA expression and overall survival (*P*=0.54, log-rank test); however, patients with tumours expressing low levels of p16/p14^ARF^ mRNA (<0.1) were found to have a significantly better event-free survival than patients with tumours expressing very high levels of p16/p14^ARF^ mRNA (⩾0.1; *P*=0.046, log-rank test; Figure 3C).

Hypermethylation of the p16 promoter was not observed in any of the 35 tumours examined; tumours with homozygous deletion of *CDKN2A* were omitted from the analysis (Figure 2B).

### Expression of p16 protein in primary ESFT

**Figure 4:**
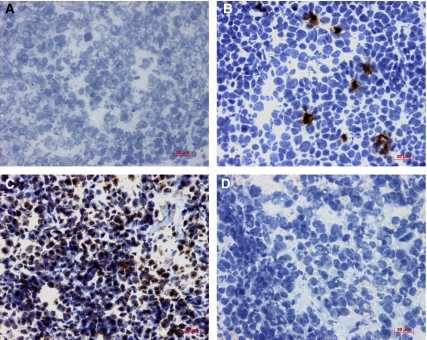
Expression of p16 protein detected by immunohistochemistry. Immunohistochemistry was performed on frozen primary ESFT (*n*=37) and p16 protein detected using the CSA System. Tumours were negative (**A**), expressed in focal hotspots (**B**) or throughout the tumour (**C**). Homozygous deletion of *CDKN2A* correlated with loss of p16 protein expression in 3/4 tumours. Immunohistochemistry performed in the absence of primary antibody controlled for nonspecific binding of the primary antibody (**D**). Magnification × 400.

Immunohistochemistry for p16 protein was performed on 37 primary ESFT. p16 was not detected in 3/4 tumours that were homozygously deleted for *CDKN2A* (Figure 4A). In one tumour (36/05) p16 was focally expressed in 23% of tumour cells. In those tumours that were p16 wild type, p16 was not expressed in 6/33 tumours.

p16 protein expression was nuclear and cytoplasmic, and in the majority of tumours (*n*=26) restricted to focal hotspots within the tumour (Figure 4B). Within these hotspots p16 was expressed within 1–23% of cells. These tumours included 3 with high p16/p14^ARF^ mRNA. Two tumours expressed high levels of p16 protein throughout the tumour; in tumour 38/01 25% of cells expressed p16 and in tumour 15/05 47% of cells expressed p16 (Figure 4C). Both these tumours had high levels of p16/p14^ARF^ mRNA. Protein expression of p16 was not prognostically significant of overall (*P*=0.78) or event-free (*P*=0.70) survival in the study group. There was no correlation between p16 protein and p16/p14^ARF^ mRNA expression.

## DISCUSSION

The frequency of homozygous deletions of *CDKN2A* detected by MLPA in ESFT cell lines (44%) and primary tumours (10%) is consistent with the literature using more established methods including Southern blot (Kovar *et al*, 1997; Tsuchiya *et al*, 2000; Wei *et al*, 2000), PCR (Tsuchiya *et al*, 2000), and fluorescent *in situ* hybridisation (Huang *et al*, 2005). Using MLPA it was also possible to detect co-deletion of the cell-cycle regulatory genes *CDKN2B* (encoding p15) and *CDKN2A* exon 1*β* (encoding p14^ARF^). We therefore conclude that MPLA is a robust, low-cost, rapid, high-throughput method to analyse the status of multiple genes in frozen primary tumour; it is not reliably informative in paraffin-embedded material (results not shown). Most importantly for precious tumour samples, the amount of nucleic acid required for MLPA is small (∼20 ng of DNA, equivalent to approximately 3000 cells or 6000 single copy target sequences). However, it is essential that DNA is isolated from a pure tumour cell population, as MLPA provides an average copy number per cell so contaminating normal cells will influence the results. The complexity of the tumour genome must also be taken in to consideration, as any abnormal copy number of control probe target sequences will result in the allocation of an inaccurate test probe sequence copy number. Therefore it is advisable to confirm the suitability of the internal control probe data set for a group of tumours. For example, in this study of ESFT, where gain of chromosome 8 is a common event (Armengol *et al*, 1997; Tarkkanen *et al*, 1999; Hattinger *et al*, 2002), the two control probes on chromosome 8 were excluded. The main advantage of MLPA over PCR-based techniques is the ability to identify single copy deletions.

No correlation was observed between homozygous deletion of *CDKN2A* and overall survival, event-free survival or the presence of metastatic disease at diagnosis. Furthermore, deletion of one copy of *CDKN2A* (5% of primary ESFT) was rare. These observations are in contrast to previous studies in ESFT. In a study of 24 ESFT, patients with tumours that had a mutation/deletion of *p16* (4/24; 17%) had a worse event-free survival than those without (*P*=0.019; Tsuchiya *et al*, 2000), and in a second study, patients with deletions of *p16* (7/39; 18%) were shown to have a worse disease-specific survival than those without (*P*=0.001; Wei *et al*, 2000). In both these studies, deletions were detected by Southern blot of total DNA from tumours compared to MLPA analysis of DNA extracted from isolated tumour cells. Using MLPA, homozygous deletion of the *CDKN2A* gene was observed in four primary ESFT; this correlated with loss of p16/p14^ARF^ mRNA expression. In the two tumours with a single gene copy loss p16/p14^ARF^ mRNA was expressed, suggesting that the remaining copy was not mutated. In all the remaining tumours p16/p14^ARF^ was expressed, although the level of expression was heterogeneous. This heterogeneity could not be attributed to hypermethylation of the p16 promoter; whether hypermethylation of the p14^ARF^ promoter is an important mechanism of downregulating p14^ARF^ expression remains to be seen. Other studies have reported p16 promoter hypermethylation in ESFT to be rare; 1/24 (4%; Tsuchiya *et al*, 2000) and 2/19 (10%; Lopez-Guerrero *et al*, 2001). Heterogeneity of expression might therefore be effected through expression of wild-type *p53*, which can downregulate transcription from the *p14*^*ARF*^ and *p16* promoter (Robertson and Jones, 1998), pRb hyperphosphorylation (Chatterjee *et al*, 2004) or overexpression of the *bmi-1* gene (Jacobs *et al*, 1999). Homozygous and single copy deletions of the *MTAP* gene were observed in ESFT cell lines and primary tumour; homozygous deletions (but not single copy deletions) have previously been described with similar frequency to this study (Huang *et al*, 2005). The *MTAP* gene was always co-deleted with *CDKN2A*, consistent with previous data (Jacobs *et al*, 1999).

Patients with tumours that expressed high levels of p16/p14^ARF^ mRNA (assessed by primers that amplified *CDKN2A* exons 2 and 3) had a significantly worse event-free survival than patients with tumours that expressed low levels of p16/p14^ARF^ (*P*=0.046, log-rank test). This is unexpected as one might predict that reduced levels of p16 would result in deregulated activation of cyclin-dependent kinase 4/6 resulting in uncontrolled cell-cycle transition through the Rb pathway, leading to a higher tumour growth rate. However, high levels of p16 mRNA expression have been related to a shorter event-free survival in acute lymphoid leukaemia (Mekki *et al*, 1999), breast cancer (Hui *et al*, 2000) and neuroblastoma (Omura-Minamisawa *et al*, 2001), and loss of p16 protein expression has previously been associated with the presence of metastatic disease at diagnosis in primary ESFT (*P*=0.026, *n*=20; Maitra *et al*, 2001). Interestingly, we found no correlation between p16 protein expression and outcome. In contrast to studies of p16 mRNA, previous studies have reported overexpression of p16 protein to be predictive of improved prognosis in oropharyngeal squamous-cell cancer (Weinberger *et al*, 2004), vulvar carcinoma (Knopp *et al*, 2004) and colorectal carcinoma (Zhao *et al*, 2003; Cui *et al*, 2004), whereas high levels of p16 protein expression have been associated with shorter event-free survival in prostate cancer (Lee *et al*, 1999). Whether these inconsistencies reflect tumour-specific roles of p16, or differences in the prognostic significance of mRNA and protein remain to be seen. In this study, expression of p16 protein did not correlate with p16/p14^ARF^ mRNA levels; however, homozygous deletion of the *CDKN2A* gene resulted in loss of p16 protein in 3/4 cases. In the remaining case, the majority of tumour cells were negative for p16 expression; however, a small clone of cells expressed p16 protein, and we predict this clone of cells must have been absent in adjacent tumour sections that were analysed by MLPA for *CDKN2A* gene status. p16 protein and mRNA levels have previously been shown not to correlate in a study of patients with adult T-cell leukaemia; tumours with high levels of p16 mRNA expression were shown to lack expression of p16 protein and showed a significantly shorter survival than patients with tumours that expressed p16 protein (Takasaki *et al*, 2003). This discrepancy may reflect the rapid post-translational degradation of p16 protein, which requires further investigation. It will also be important to investigate the expression and prognostic significance of p14^ARF^ mRNA and protein; previous studies have shown no correlation between p14^ARF^ protein expression and outcome in ESFT (Maitra *et al*, 2001) although in squamous-cell carcinoma loss of expression is reported to predict a worse overall and event-free survival (Kwong *et al*, 2005).

In summary, we have shown high levels of p16/p14^ARF^ mRNA in tumours taken at diagnosis predict a significantly worse event-free survival in patients with ESFT. However, p16 protein expression was not prognostically significant. The disparity in these results may be explained by the complex interaction of multiple cell-cycle regulatory proteins on tumour proliferation and progression. The inconsistency of data in the literature on the profile and prognostic significance of p16 and p14^ARF^ gene status, mRNA and protein expression in cancer emphasises the importance of investigating these and interacting cell-cycle regulatory genes in the same clinical samples, according to standardised methods, in a prospective clinical outcome study. High-throughput methods such as MLPA may facilitate such analyses.

## Figures and Tables

**Table 1:** Summary of the status of the genes located to chromosome 9p21 in ESFT and neuroblastoma cell lines

	**Cell line**											
**Probe**	**TC-32**	**RDES**	**TTC-466**	**SKES1**	**GROH1**	**SK-N-MC**	**A673**	**STAET1**	**STAET11**	**IMR-32**	**SH-SY-5Y**	**SK-N-SH**
*TEK*	+	+	+	±	+	+	±	+	+	+	+	+
*ELAVL2*	±	±	+	±	+	+	±	+	+	+	+	+
p15 promotor/exon 1	±	±	+	±	+	+	−	−	−	+	+	+
p15 exon 1	±	±	+	±	+	+	−	−	−	+	+	+
p15 intron	±	±	+	±	+	+	−	−	−	+	+	+
0.5 kb upstream of p14^ARF^	±	±	+	±	+	+	−	−	−	+	+	+
p14^ARF^ exon 1*β*	±	±	+	±	+	+	−	−	−	+	+	+
Intron between p14^ARF^ and p16 exon 1	±	±	+	±	+	+	−	−	−	+	+	+
Second intron probe between p14^ARF^ and p16 exon 1	±	±	+	±	+	+	−	−	−	+	+	+
p16 exon 1	−	±	+	±	+	+	−	−	−	+	+	+
p16 exon 2	−	±	+	±	+	+	−	−	−	+	+	+
p16 exon 3	−	±	+	±	+	+	−	−	−	+	+	+
MTAP probe 3; end of *MTAP* gene	−	±	+	±	+	+	±	+	+	+	+	+
MTAP probe 2	−	±	+	±	+	+	±	+	+	+	+	+
MTAP probe 1; start of *MTAP* gene	±	±	+	±	+	+	±	+	+	+	+	+
*KIAA1354*	+	±	+	±	+	+	±	+	+	+	+	+
*IFNW1*	+	±	+	±	+	+	±	+	+	+	+	+
*IFNB1*	+	±	+	±	+	+	±	+	+	+	+	+
MLLT3 probe 2	+	±	+	±	+	+	±	+	+	+	+	+
MLLT3 probe 1	+	±	+	±	+	+	±	+	+	+	+	+
*FLJ00026*	+	±	+	±	+	+	+	+	+	+	+	+

ESFT=Ewing's sarcoma family of tumours, MLPA=multiplex ligation-dependent probe amplification.

DNA was extracted from the cell lines and subjected to MLPA using the 9p21 kit. +=probe copy number wild type (two copies present),−=complete deletion of target sequence, ±=hemizygous deletion (one copy present).

**Table 2:** Summary of the status of the genes located to chromosome 9p21 in ESFT where abnormalities were identified

	**Tumour**					
**Probe**	**04/03**	**07/01**	**27/02**	**72/03**	**29/99**	**36/05**
*TEK*	+	+	+	+	+	+
*ELAVL2*	+	+	+	+	+	+
p15 promotor/exon 1	+	−	+	+	±	−
p15 exon 1	+	−	+	+	±	−
p15 intron	+	−	−	+	±	−
CpG island at p14^ARF^ promotor	+	−	−	+	±	−
0.5 kb upstream of p14^ARF^	+	−	−	+	±	−
p14^ARF^ exon 1*β*	+	−	−	+	±	−
Intron between p14^ARF^ and p16 exon 1	+	−	−	+	±	−
Second intron probe between p14^ARF^ and p16 exon 1	+	−	−	+	±	−
p16 exon 1 *α*	+	−	−	±	±	−
p16 exon 1 *α*	+	−	−	±	±	−
p16 exon 2	−	−	−	+	±	−
p16 exon 3	−	−	−	+	±	−
MTAP probe 3; end of *MTAP* gene	−	−	+	+	±	−
MTAP probe 2	−	−	+	+	±	−
MTAP probe 1; start of *MTAP* gene	+	−	+	+	±	−
*KIAA1354*	+	+	+	+	+	±
*IFNW1*	+	+	+	+	+	±
*IFNB1*	+	+	+	+	+	±
MLLT3 probe 2	+	+	+	+	+	+
MLLT3 probe 1	+	+	+	+	+	+
*FLJ00026*	+	+	+	+	+	+

ESFT=Ewing's sarcoma family of tumours, LCM=laser-capture micro-dissection system, MLPA=multiplex ligation-dependent probe amplification.

DNA was extracted from tumour cells isolated by LCM from 42 primary ESFT and subjected to MLPA using the 9p21 kit. Wild-type copy number of all of the genes tested was observed in 36 ESFT (data not shown), six ESFT exhibited homozygous or single copy deletion of one or more probes. += probe copy number normal (two copies present),–= complete deletion of target gene, ±=hemizygous deletion (one copy present).
